# Complete Genome Sequence of a Novel Human Betapapillomavirus, HPV-159

**DOI:** 10.1128/genomeA.00298-13

**Published:** 2013-05-30

**Authors:** Boštjan J. Kocjan, Lea Hošnjak, Katja Seme, Mario Poljak

**Affiliations:** Institute of Microbiology and Immunology, Faculty of Medicine, University of Ljubljana, Ljubljana, Slovenia

## Abstract

A novel human papillomavirus (HPV), now officially recognized as HPV-159, isolated from an anal swab, was fully cloned, sequenced, and genetically characterized. HPV-159 has a genomic organization that is typical of cutaneotrophic HPV types, and it belongs to the genus *Betapapillomavirus.*

## GENOME ANNOUNCEMENT

Human papillomaviruses (HPVs) are a diverse family of small double-stranded DNA viruses, etiologically linked with various skin and mucosal epithelial lesions. Based on their nucleotide similarities in the L1 gene, HPVs are classified into genera, species, and types (1). Currently, >155 HPV types have been completely characterized, and the majority are placed into three genera: *Alphapapillomavirus*, predominantly found in anogenital lesions, and *Betapapillomavirus* and *Gammapapillomavirus*, typically isolated from skin and hair follicle specimens and recently from other locations, such as the oral cavity (1–3). Here, we report the complete genomic sequence of a novel HPV type obtained from an immunocompetent 36-year-old male participating in an ongoing study of the distribution of cutaneotrophic HPV types in the anal canal of Slovenian men who have sex with men.

A complete viral genome was preamplified using phi29 DNA polymerase (4) and then was PCR amplified using two overlapping primer sets: SIBX8F-LR(62) (5′-ACCTGCATTCATAGCATTAATCTGTG-3′) and SIBX8R-LR(59) (5′-AGATGCTGTGGAGCCTACAGAA-3′), and CP62 and CP70a (5), resulting in 7,348-bp and 761-bp fragments, respectively. Both amplicons were cloned using a TOPO XL PCR cloning kit (Invitrogen, Carlsbad, CA) and sequenced using a primer-walking strategy at Microsynth AG (Balgach, Switzerland). The complete viral genome was assembled and characterized using Vector NTI Advance 11 software (Invitrogen). Phylogenetic analysis was performed with the maximum-likelihood algorithm using the MEGA5 software (6). The two reference clones, covering the full genome of HPV-159, were deposited in July 2012 in the Reference Centre for Papillomaviruses, Heidelberg, Germany, where their sequences were independently confirmed, and the genotype was officially named in September 2012.

The complete genome is 7,443 bp in length, with a G+C content of 40.6%, and it contains five early (E1, E2, E4, E6, and E7) and two late (L1 and L2) genes, but no E5 open reading frame (ORF). The long control region (LCR) of 411 bp is positioned between the L1 and E6 genes and contains two consensus palindromic E2-binding sites (ACC-N_6_-GGT), two putative TATA boxes (TATAAA) of E6 promoter, and the polyadenylation site (AATAAA) for L1 and L2 transcripts (7, 8). The putative E6 protein exhibits two conserved zinc-binding domains of CxxC(x) _29_CxxC, separated by 36 amino acids (9). The putative E7 protein contains one slightly modified zinc-binding domain, CxC(x) _29_CxxC, and the standard motif LxCxE for binding to the cell retinoblastoma protein (7, 9). E1 codes for the largest viral protein, which contains 606 amino acids; the ATP-binding site (GPPDTGKS) of the ATP-dependent helicase is present in the carboxy-terminal part of E1 (10). The putative E4 ORF contains a start codon and completely overlaps the E2 ORF; the E4 protein typically contains a high proline content (19.7%). HPV-159 belongs to the cutaneotrophic genus *Betapapillomavirus*, species Beta-2, and it is most closely related to HPV-9 (similarity in L1, 79.5%). In conclusion, the genetic characterization of HPV-159 adds to the present repertoire of betapapillomaviruses and indicates a broader spectrum of epithelial tropism than has been appreciated previously for HPV types from the genus *Betapapillomavirus*.

### Nucleotide sequence accession number.

The complete genome sequence of HPV-159 is available in the EMBL, GenBank, and DDBJ databases under the accession no. HE963025.
